# DNA Methylation in Noncancerous Liver Tissues as Biomarker for Multicentric Occurrence of Hepatitis C Virus–Related Hepatocellular Carcinoma

**DOI:** 10.1016/j.gastha.2022.02.016

**Published:** 2022-05-06

**Authors:** Hiroyuki Suzuki, Hideki Iwamoto, Ken Yamamoto, Mai Tsukaguchi, Toru Nakamura, Atsutaka Masuda, Takahiko Sakaue, Toshimitsu Tanaka, Takashi Niizeki, Shusuke Okamura, Shigeo Shimose, Tomotake Shirono, Yu Noda, Naoki Kamachi, Ryoko Kuromatsu, Toru Hisaka, Hirohisa Yano, Hironori Koga, Takuji Torimura

**Affiliations:** 1 Division of Gastroenterology, Department of Medicine, Kurume University School of Medicine, Kurume, Japan; 2 Department of Medical Biochemistry, Kurume University School of Medicine, Kurume, Japan; 3 Department of Surgery, Kurume University School of Medicine, Kurume, Japan; 4 Department of Pathology, Kurume University School of Medicine, Kurume, Japan

**Keywords:** Hepatocellular Carcinoma, DNA Methylation, Hepatitis C Virus, Multicentric Occurrence

## Abstract

**Background and Aims:**

Hepatitis C virus (HCV)-related hepatocellular carcinoma (HCC) progresses with a highly multicentric occurrence (MO) even after radical hepatectomy. Despite several efforts to clarify the pathogenesis of MO, the underlying molecular mechanism remains elusive. The aim of this study was to evaluate alterations in DNA methylation in noncancerous liver tissues in the MO of HCC.

**Methods:**

A total of 203 patients with HCV-related HCC who underwent radical hepatectomy at our hospital between January 2008 and January 2012 were recruited. We defined a group of nonearly recurrence of HCC (NR) for ≥3 years after radical hepatectomy and a group of early recurrence of HCC (ER) with MO within 2 years after radical hepatectomy.

**Results:**

Three patients each were selected in the NR and ER groups in the first set, and 13 patients in the NR group and 17 patients in the ER group were selected in the second set. Genome-wide DNA methylation profiles were obtained from noncancerous liver tissues using a Human Methylation 450 BeadChip, and the differences between the groups were analyzed for each set. After excluding single nucleotide polymorphism-associated methylation sites and low-call sites, 401,282 sites were assessed using a generalized linear model without any adjustments. Nine gene regions, *APBB1P*, *CLSTN3*, *DLG5*, *IRX5*, *OAS1*, *SOX12*, *SNX19*, *TENM2*, and *TRIM54*, exhibiting a significant difference (*P* < .001) in DNA methylation levels were identified in the common direction between the 2 analysis sets.

**Conclusion:**

Alterations in DNA methylation of 9 genes in noncancerous liver tissues appear to be involved in MO after radical hepatectomy for HCV-related HCC.

## Introduction

Hepatocellular carcinoma (HCC) is the most common primary liver cancer and the third leading cause of cancer-related deaths worldwide.^1^^,^^2^ HCC typically occurs in the setting of persistent hepatitis or cirrhosis secondary to hepatitis B virus and hepatitis C virus (HCV) infection, alcoholism, or nonalcoholic steatohepatitis.^2^ Epidemiologic evidence has indicated that persistent infection with HCV is a major risk factor for developing HCC, and cirrhosis is the main cause of HCV-related HCC; the core protein of HCV has also been considered to be directly involved in hepatocarcinogenesis.^3^ The high recurrence rate of HCV-related HCC is dependent on its characteristics, such as intrahepatic metastasis and multicentric occurrence (MO).^1^ Even after radical treatment, such as hepatectomy and radiofrequency ablation, the low disease-free survival rates of HCV-related HCC (5 years, 24%) result in high mortality rate and limited prognosis.^4^^,^^5^ Therefore, identifying patients at high risk of HCC recurrence after radical treatment is important. One of the mechanisms that cause MO in HCV-related HCC, even after sustained virologic response (SVR), is the epigenetic alteration of hepatocytes in the liver caused by chronic hepatitis.^6^^,^^7^

In the last few decades, epigenetic alterations have been shown to be almost universal among different types of cancers and have become increasingly important for understanding human malignancies.^8^ DNA methylation, a major epigenetic alteration, regulates the gene transcription and stability without affecting the DNA sequence.^8^ Aberrant accumulation of DNA methylation, caused by a long-term exposure to carcinogens and chronic inflammation, is known to form a carcinogenic field in a variety of malignancies and to play an important role in carcinogenesis; for example, methylation within the promoter region of tumor-suppressor genes causes their silencing and methylation within the gene itself may trigger mutational events.^9^ Genome-wide DNA methylation profiling of various types of cancers, including HCC, has shown that substantial alteration in DNA methylation plays an important role in the setting of carcinogenesis.^10^ Aberrant methylation of promoter CpG islands in several genes has been reported to be a possible biomarker for HCV-related HCC development; however, the detailed mechanisms of the relationship between MO and DNA methylation have not been clarified.^11^, ^12^, ^13^ Furthermore, there are no comprehensive reports comparing the histologic DNA methylation levels in noncancerous liver tissues between cases with and without early MO after radical hepatectomy.

The aim of this study was to verify whether alterations in histologic DNA methylation in noncancerous liver tissues of HCV-related HCC are involved in the MO.

## Methods

### Patients

**Figure 1:**
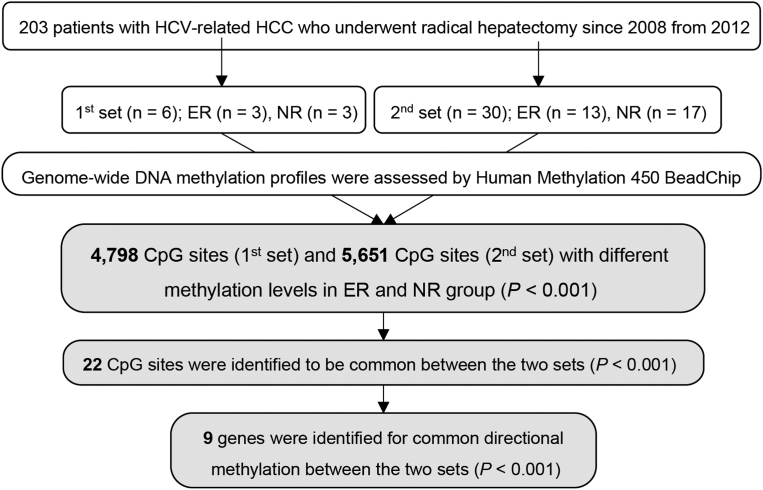
Flowchart of this study.

Tissue samples from 203 patients with HCV-related HCC were obtained by radical hepatectomy at our hospital between January 2008 and January 2012. The exclusion criteria were as follows: (1) HCC recurrence pattern exhibiting characteristics of intrahepatic metastasis (recurrent within 3 months after radical hepatectomy); (2) under systemic steroid therapy, which is known to directly affect DNA methylation^14^^,^^15^; (3) inadequate follow-up after radical hepatectomy; and (4) HCC previously treated with systemic chemotherapy. Eventually, a total of 36 patients were selected and were assigned to 2 groups: nonearly recurrence group (NR), patients without HCC recurrence, >3 years after hepatectomy; and early recurrence group (ER), patients with multicentric recurrence of HCC within 3 years after hepatectomy. We performed analysis of the 2 sets; in the first set experiments, 3 cases each from the NR and ER groups were selected, and 3 different tissues were collected from each patient. At least 9 tissues from each group were evaluated. In the second set of experiments, 13 and 17 cases from the NR and ER groups, respectively, were selected. In the second set, one tissue sample was collected from each patient (Figure 1). Noncancerous liver tissues were taken from the resected tissues when performed radical hepatectomy. These tissues were immediately frozen in liquid nitrogen and kept at −80 °C until the following DNA extracting preparation.

### DNA Methylation Chip Assay

After extracting DNA from noncancerous liver tissues, DNA methylation levels of about 470,000 CpG methylation sites located on the autosome were determined using an Illumina Human Methylation 450 BeadChip (Illumina, San Diego, CA) according to the manufacturer’s instructions. For 401,282 CpG methylation sites, excluding CpG sites related to single nucleotide polymorphisms or sites with poor data acquisition rate, the differences in DNA methylation levels between the NR and ER groups were analyzed using the generalized linear model without any adjustments.^16^ Gene expression levels in the liver were obtained from the Genotype-Tissue Expression project.^17^

### Statistical Analysis

Data are expressed as numbers or medians. Differences between the 2 groups were analyzed using the Wilcoxon signed-rank test. *P* values <.05 and <.001 were considered to indicate statistically significant and extremely significant differences, respectively. The difference in DNA methylation levels was tested using a generalized linear model, and significant differences between the ER and NR groups were determined after Bonferroni correction. Data analyses were performed using R Statistical Software (version 2. 15. 2; R Foundation for Statistical Computing, Vienna, Austria).

### Ethics Statement

This study was performed in accordance with the principles of the Declaration of Helsinki and was approved by the ethical committee of the Kurume University School of Medicine (Ethical code: 144) and the Institutional Review Board of Kyushu University (Ethical code: 538-00).

## Results

### Patient Characteristics

**Figure 2:**
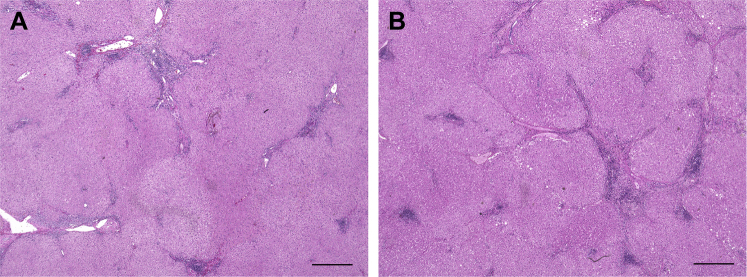
Representative histopathological findings. Representative pathologic images of the noncancerous liver tissues from (A) NR and (B) ER groups (hematoxylin and eosin staining). Scale bars represent 500 μm.

In the first set, the median age was 67.9 years, and 66.7% of the patients were male (Table 1). In the second set, the median age was 72.2 years, and 63.3% of the patients were male (Table 2). There were no significant differences with respect to the presence of diabetes, Child-Pugh score, prevalence of SVR, tumor characteristics (such as serum tumor markers, tumor number, and tumor differentiation), and histopathological findings between the NR and ER groups (Tables 1 and 2); whereas, in the second set, the patients in the ER group were younger than those in the NR group (68.4 vs 75.2; *P* = .032; Table 2). In the first set, the median recurrence-free observation period was 1390 (range, 1285–1421) days for the NR group and 419 (range, 370–469) days for the ER group (Table 1). The median recurrence-free observation period was 1449 (range, 1111–2474) days for the NR group and 784 (range, 270–914) days for the ER group in the second set (Table 2). The representative histologic image of the cases from ER and NR groups is shown in Figure 2, whose degree of HCV-related cirrhosis was F2A2.

**Table 1:** Baseline Clinical and Tumor Characteristics for the First Set

Clinical characteristics	Total (n = 6)	NR (n = 3)	ER (n = 3)	*P* value
Age (y)	67.9	63.1	71.6	.067
Gender, male/female	4/2	2/1	2/1	1.000
Diabetes mellitus +/−	4/2	1/2	3/0	.083
Child-Pugh score 5/6	4/2	2/1	2/1	1.000
Liver damage A/B	4/2	2/1	2/1	1.000
AST (IU/L)	73.5	42.7	104.3	.140
ALT (IU/L)	33.7	32.0	35.3	.819
Total bilirubin (mg/dL)	0.74	0.66	0.79	.56
Platelet (×10^3^/μL)	12.7	12.9	12.4	.865
SVR +/−	2/4	0/3	2/1	.051
Tumor characteristics				
TNM classification	0/4/2	2/1/0	2/1/0	1.000
Number 1/2/3	4/1/1	1/1/1	3/0/0	.148
Tumor size (mm)	25.2	29.3	21.7	.200
Alpha-fetoprotein (ng/mL)	462.8	30.0	903.3	.371
DCP (mAU/mL)	1140.8	2258.3	23.3	.372
Pathological findings				
Fibrosis 1/2/3/4	1/0/2/3	1/0/0/2	0/0/2/1	.101
Activity 1/2	0/6	0/3	0/3	1.000
Differentiation well/moderate/poor	2/4/0	1/2/0	1/2/0	1.000
Macroscopic classification SN/SNEG/CMN/others	3/0/1/2	1/0/1/1	2/0/0/1	.422
Clinical time course				
Median recurrence-free observation period (d)	1599	1390	419	

*P* values were obtained by comparing between the NR and ER groups.

AFP, alpha-fetoprotein; ALT, alanine aminotransferase; AST, aspartate aminotransferase; CMN, confluent multinodular type; DCP, des-γ-carboxy prothrombin; SN, simple nodular type; SNEG, simple nodular type with extranodular growth.

**Table 2:** Baseline Clinical and Tumor Characteristics for the Second Set

Clinical characteristics	Total (n = 30)	NR (n = 13)	ER (n = 17)	*P* value
Age (y)	72.2	68.4	75.2	.032
Gender male/female	19/11	6/7	13/4	.088
Diabetes mellitus +/−	19/11	6/7	13/4	.088
Child-Pugh score 5/6	20/10	7/6	12/5	.346
Liver damage A/B	18/12	9/4	9/8	.301
AST (IU/L)	51.1	51.8	50.6	.940
ALT (IU/L)	53.1	51.7	54.0	.888
Total bilirubin (mg/dL)	0.76	0.82	0.71	.360
Platelet (×10^3^/μL)	14.7	15.4	14.1	.500
SVR +/−	4/26	3/10	1/16	.170
Tumor characteristics				
TNM classification	5/20/5	2/11/0	3/9/5	.084
Number 1/2/3	23/6/1	12/1/0	11/5/1	.199
Tumor size (mm)	28.7	30.3	27.8	.650
Alpha-fetoprotein (ng/mL)	167.0	166.6	172.8	.973
DCP (mAU/mL)	1140.8	2258.3	23.3	.512
Pathological findings				
Fibrosis 1/2/3/4	7/6/6/11	4/5/1/3	3/1/5/8	.051
Activity 1/2	6/24	3/10	3/14	.713
Differentiation well/moderate/poor	3/23/4	2/11/0	1/12/4	.299
Macroscopic classification SN/SNEG/CMN/others	21/5/1/3	9/2/0/2	12/3/1/1	.693
Clinical time course				
Median recurrence-free observation period (d)	1116	1449	784	

*P* values were obtained by comparing between the NR and ER groups.

ALT, alanine aminotransferase; AST, aspartate aminotransferase; CMN, confluent multinodular type; DCP, des-γ-carboxy prothrombin; SN, simple nodular type; SNEG, simple nodular type with extranodular growth.

### DNA Methylation Profiling Across the Genome

**Figure 3:**
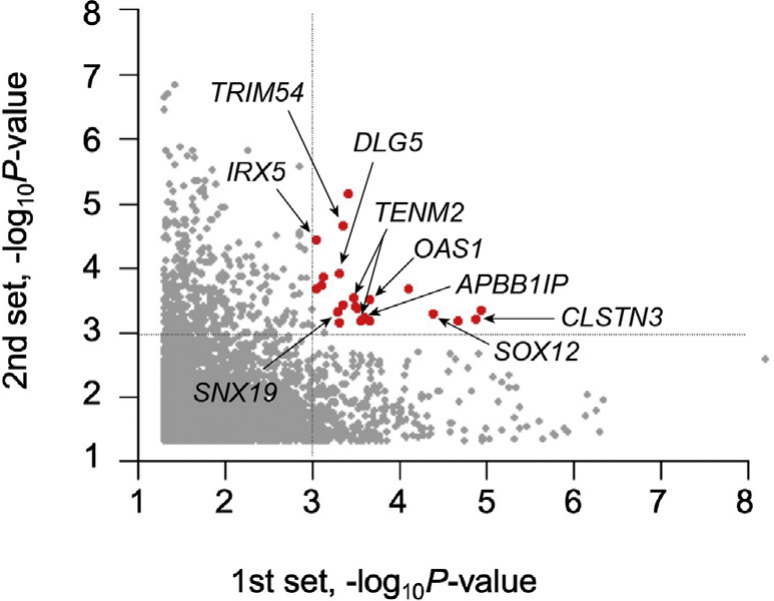
Dots plots for differences in CpG methylation sites. Common significant differences between the 2 sets. Red plots represent the 22 CpG sites, which showed commonly significant differences between the 2 groups (*P* < .001).

**Figure 4:**
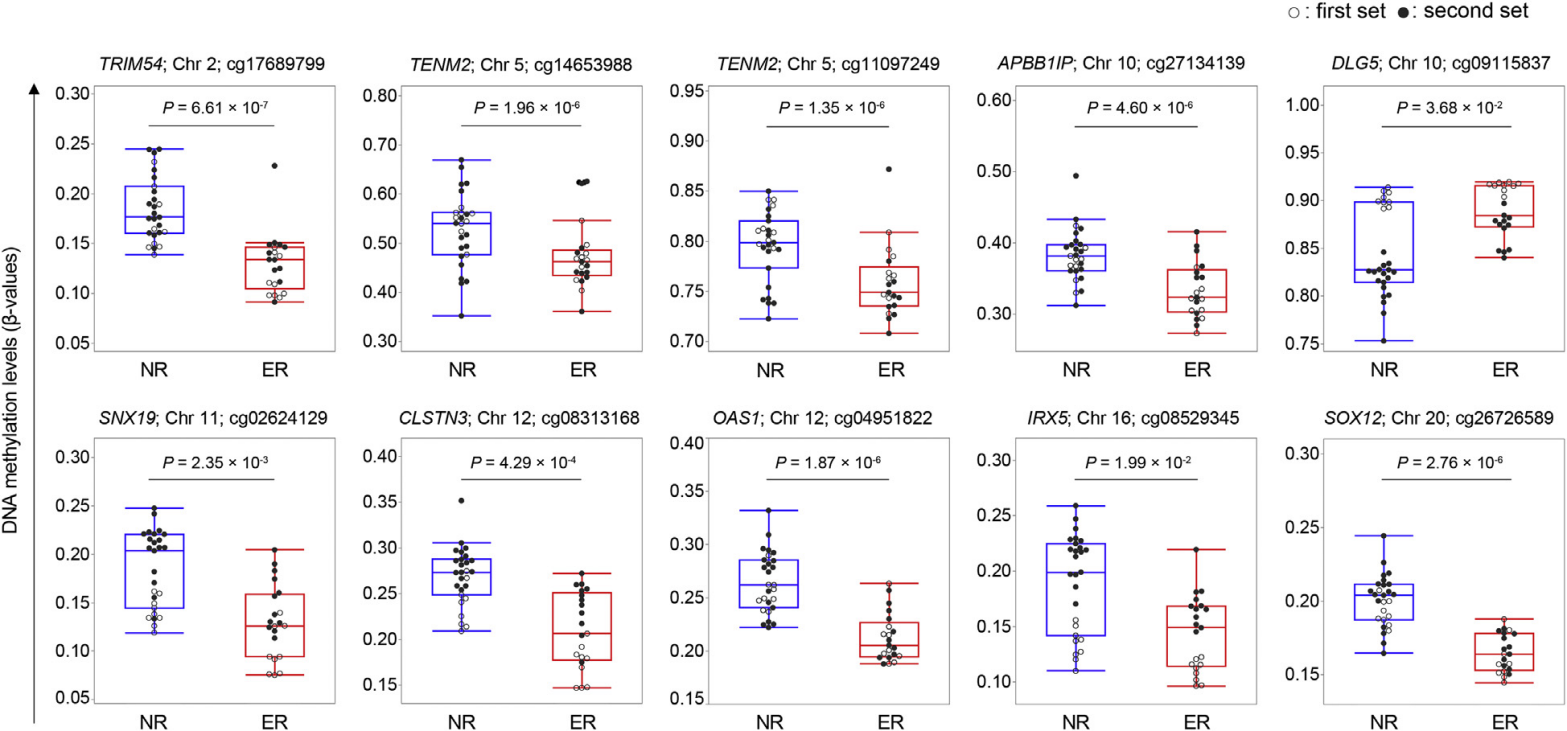
DNA methylation levels at 10 CpG sites. White dots represent patients in the first set, and black dots represent patients in the second set.

Among the ER and NR groups, 4798 and 5651 CpG methylation sites were significantly different in the first and second sets, respectively. Among these CpG methylation sites, 22 were found to have significant changes in common between the 2 sets (Figure 3). Excluding genes with different directional methylation (hypo vs hyper) between the first and second sets, 10 CpG methylation sites and 9 genes were identified for common directional methylation changes between the 2 sets (Table 3). The methylation level of each gene is shown in Figure 4. In the methylation analysis, we observed a potential functional role of hypomethylation in 8 HCV-related HCC that affected their genes (*TRIM54*, *TENM2*, *APBB1IP*, *SNX19*, *CLSTN3*, *OAS1*, *IRX5*, and *SOX12*) between the NR and ER groups in common between the 2 sets (Table 3). From the Genotype-Tissue Expression project,^15^ the highest expression level in the liver was observed for *CLSTN3* (25.84 transcripts per million), followed by that for *APBB1IP* (18.84 transcripts per million) and *SNX19* (13.12 transcripts per million; Table 3).

**Table 3:** Ten CpG Cites With the Same Direction of DNA Methylation and Significantly Different Between the 2 Sets

Chromosome	Position	CpG site	Gene	First set		Second set		Combine		Gene expression in the liver (TPM)
				Beta	*P* value	Beta	*P* value	Beta	*P* value	
2	27,529,540	cg17689799	TRIM54	−0.056	4.59 × 10^−4^	−0.055	2.20 × 10^−5^	−0.053	6.61 × 10^−7^	0.092
5	167,171,284	cg14653988	TENM2	−0.076	3.12 × 10^−4^	−0.104	4.13 × 10^−4^	−0.093	1.96 × 10^−6^	0.371
5	167,394,019	cg11097249	TENM2	−0.049	2.82 × 10^−4^	−0.049	6.47 × 10^−4^	−0.050	1.35 × 10^−6^	0.371
10	26,681,347	cg27134139	APBB1IP	−0.057	3.30 × 10^−4^	−0.055	4.01 × 10^−4^	−0.054	4.60 × 10^−6^	18.84
10	79,541,592	cg09115837	DLG5	0.013	5.08 × 10^−4^	0.043	1.17 × 10^−4^	0.027	3.68 × 10^−2^	2.232
11	130,786,230	cg02624129	SNX19	−0.041	5.20 × 10^−4^	−0.047	4.94 × 10^−4^	−0.040	2.35 × 10^−3^	13.12
12	7,315,531	cg08313168	CLSTN3	−0.065	1.35 × 10^−5^	−0.039	6.32 × 10^−4^	−0.045	4.29 × 10^−4^	25.84
12	113,345,598	cg04951822	OAS1	−0.045	2.22 × 10^−4^	−0.048	3.11 × 10^−4^	−0.046	1.87 × 10^−6^	7.165
16	54,971,700	cg08529345	IRX5	−0.024	9.07 × 10^−4^	−0.042	3.69 × 10^−5^	−0.031	1.99 × 10^−2^	0.018
20	306,364	cg26726589	SOX12	−0.032	4.15 × 10^−5^	−0.029	4.99 × 10^−4^	−0.029	2.76 × 10^−6^	3.356

TPM, transcripts per million.

## Discussion

In the present study, we observed that 9 gene regions, *viz.*, *APBB1P*, *CLSTN3*, *DLG5*, *IRX5*, *OAS1*, *SOX12*, *SNX19*, *TENM2*, and *TRIM54*, exhibited significant differences in the DNA methylation levels (*P* < .001) in the common direction between the ER and NR groups in the 2 analysis sets.

Eight of these 9 genes had higher DNA methylation levels in the ER group compared with that in the NR group. In general, it is known that the higher the number of methylation sites contributes worse prognosis in patients with cancer. Our results are consistent with those presented in a recent report on “proliferation class” of HCC, which is characterized as a clinically aggressive tumor.^18^ We evaluated the correlation between MO and DNA methylation by examining the function of genes whose DNA methylation levels were lower in the ER group compared with those in the NR group.

*TRIM54*, a member of the tripartite motif family, is thought to be involved in several biological processes, such as carcinogenesis, cellular growth, and cellular differentiation.^19^ Recently, using RNA-sequencing data of 292 HCC patients from The Cancer Genome Atlas, *TRIM54* was reported to be one of the vascular invasion-associated 14-gene signature to predict the prognosis in patients with HCC.^20^ In addition, *TRIM54* has been reported to affect the expression of p62/IMP2, which is an essential regulator of Wnt signaling pathways and plays an important role in the progression and metastasis of HCC.^21^

In patients with malignant ovarian cancer, decreased *TENM2* expression is significantly associated with shortened overall survival.^22^ It has been reported that compared with primary cultured human hepatic stellate cells, alcohol-injured human hepatic stellate cells exhibit downregulation of the expression of *TENM2*.^23^ As these conditions can precede HCC, the presence of early genetic alterations might be considered as an initiating event implicated in carcinogenesis. In line with these findings, we show that hypomethylation of *TENM2* is associated with the early recurrence of HCC.

*ABPP1IP*, identified as a Rap1-binding protein that is important in the activation of leukocyte integrin, is known to be involved in the infiltration ability of leukocytes.^24^ Although there are no reports on the relationship between *APBB1IP* and HCC or HCV infection, several studies have revealed the relationship between *APBB1IP* and malignancies. In a study on clear cell renal cell carcinoma, circular-*APBB1IP* was reported to be a prognostic biomarker promoting tumor growth and invasion through the activation of ERK1/2 signals.^25^ In another study on colorectal cancer using cell-free DNA, hypermethylation of *APBB1IP* was identified as a novel candidate for cancer detection.^26^ Furthermore, in a study on gastric cancer, 19 serum proteins, including *APBB1IP*, were used to distinguish patients with and without cancer.^27^

*SNX19*, a central regulator of cell trafficking and signal transduction, has been identified as one of the genes associated with diabetes and schizophrenia.^28^ Although there is no report on the relationship between *SNX19* and HCV infection or HCC, it has been reported that 18 genes, including *SNX19*, were significantly overexpressed in thyroid oncocytic adenomas compared with their expression in controls.^29^

*CLSTN3*, a member of evolutionarily conserved synaptogenic adhesion proteins of the cadherin superfamily, was identified as a synaptic adhesion molecule that acts through presynaptic neurexins.^30^*CLSTN3* is known to regulate neural regulation of energy and bone homeostasis.^31^ *CLSTN3* was reported to be upregulated in patients with hepatocellular adenoma compared with that in patients with HCC, and *CLSTN3* was recognized to be involved with the difference in the degree of HCV-related cirrhosis (in the common term between; F2 vs F4 and F3 vs F4, F1 vs F3).^32^^,^^33^

*OAS1* is an interferon-stimulating gene that plays an important role in the innate immune response, cell differentiation, and apoptosis.^34^ Single nucleotide polymorphisms in *OAS1* are associated with susceptibility to chronic HCV infection, progression of hepatic fibrosis, hepatic necroinflammatory activity grade, and development of HCC.^35^^,^^36^ The expression of *OAS1* was negatively correlated with breast and prostate cancer progression, whereas it was positively correlated with prognosis in patients with colorectal cancer.^37^^,^^38^ Because *OAS1* is strongly associated with HCV infection and tumor prognosis, it is reasonable that there is a correlation between changes in methylation in noncancerous liver tissue and MO.

*IRX5* plays pivotal roles in normal embryonic cell patterning and in the development of malignancies.^39^ *IRX5* was reported to be upregulated in HCC tissues compared with that in adjacent normal tissues, and it promotes the proliferation of HCC cells by upregulating the expression of cyclin D1.^40^ Several studies regarding the association of *IRX5* with cancers have shown that depending on the type of cancer, *IRX5* could act both in tumor-suppressing and tumor-promoting roles via the transforming growth factor beta signaling.^41^^,^^42^ In the present study, *IRX5* was hypomethylated in the NR group compared with that in the ER group; therefore, further studies are warranted to clarify the function of *IRX5* in noncancerous tissues.

*SOX12* is a member of the sex-determining region Y–related high mobility group box family of transcription factors that is characterized by the presence of a highly conserved DNA-binding high mobility group domain. In HCC, *Sox12* is one of the cancer stem cell markers.^43^ The expression of *SOX12* is correlated with the malignancy of HCC (such as extracapsular invasion, microvascular invasion, and lymph node metastasis), and higher *SOX12* levels are associated with poorer prognosis in HCC patients (overall survival *P* = .0004, progression-free survival *P* = .0013).^44^^,^^45^ Specifically, *SOX12* activates the expression of *Twist1* and fibroblast growth factor–binding protein 1, which promote the epithelial-mesenchymal transition of HCC and increase the expression of CDK14 and insulin-like growth factor 2 mRNA-binding protein 1, defining the malignant phenotype of HCC.^44^^,^^46^ Furthermore, some cancer-related microRNAs (miR), such as miR-874 and miR-744, have been reported to directly target *SOX12* and suppress its action.^47^^,^^48^ Further elucidation of the relationship with DNA methylation in *SOX12* is desired.

In the present study, DLG5 was hypermethylated in the group with MO. It has been reported that DLG5 is downregulated in HCC, and lower DLG5 expression is associated with poor survival of patients with HCC, and that the accumulation or overexpression of DLG5 inhibits the proliferation and intrahepatic and lung metastasis of HCC cells.^49^^,^^50^

The present study had several limitations, including its single-center retrospective design and the small sample size. In addition, although DNA methylation is generally considered a stable epigenetic mark, there are possibilities of false positives and ignoring local changes. Furthermore, there were significant differences for *DLG5* and *IRX2* when the first and second sets were independently assessed (*P* < .001); however, when the 2 sets were combined, the difference became smaller and exhibited the same tendency. This may be because the methylation levels of these genes were separated in the first and second sets (Table 3). Unlike the fact that a very high SVR rate was achieved using direct-acting antivirals in clinical practice, the SVR rate was only 20% for the patients in this study.^51^ However, because epigenetic alterations remain present even after SVR obtained by interferon or direct-acting antiviral treatment, it is important to evaluate epigenetic alterations regardless of the achievement of SVR.^7^^,^^52^ The elimination of these limitations requires the accumulation of clinical cases, which could enable more accurate prediction of HCV-related HCC recurrence.

In conclusion, after radical hepatectomy of HCV-related HCC, methylation levels at particular CpG sites in DNA from noncancerous liver tissues appear to be suitable biomarkers for predicting MO of HCC. Further validation in a large cohort of patients is required.

## References

[1] Sung H., Ferlay J., Siegel R.L. (2021). Global Cancer Statistics 2020: GLOBOCAN estimates of incidence and mortality worldwide for 36 cancers in 185 countries. CA Cancer J Clin.

[2] El-Serag H.B., Rudolph K.L. (2007). Hepatocellular carcinoma: epidemiology and molecular carcinogenesis. Gastroenterology.

[3] Koike K. (2007). Hepatitis C virus contributes to hepatocarcinogenesis by modulating metabolic and intracellular signaling pathways. J Gastroenterol Hepatol.

[4] Sasaki Y., Yamada T., Tanaka H. (2006). Risk of recurrence in a long-term follow-up after surgery in 417 patients with hepatitis B- or hepatitis C-related hepatocellular carcinoma. Ann Surg.

[5] Takeishi K., Maeda T., Tsujita E. (2015). Predictors of intrahepatic multiple recurrences after curative hepatectomy for hepatocellular carcinoma. Anticancer Res.

[6] Ding X., He M., Chan A.W.H. (2019). Genomic and epigenomic features of primary and recurrent hepatocellular carcinomas. Gastroenterology.

[7] Hamdane N., Jühling F., Crouchet E. (2019). HCV-induced epigenetic changes associated with liver cancer risk persist after sustained virologic response. Gastroenterology.

[8] Baylin S.B., Jones P.A. (2011). A decade of exploring the cancer epigenome - biological and translational implications. Nat Rev Cancer.

[9] Wajed S.A., Laird P.W., DeMeester T.R. (2001). DNA methylation: an alternative pathway to cancer. Ann Surg.

[10] Laird P.W. (2010). Principles and challenges of genome-wide DNA methylation analysis. Nat Rev Genet.

[11] Calvisi D.F., Ladu S., Gorden A. (2007). Mechanistic and prognostic significance of aberrant methylation in the molecular pathogenesis of human hepatocellular carcinoma. J Clin Invest.

[12] Nishida N., Kudo M., Nagasaka T. (2012). Characteristic patterns of altered DNA methylation predict emergence of human hepatocellular carcinoma. Hepatology.

[13] Kanda T., Goto T., Hirotsu Y. (2019). Molecular mechanisms driving progression of liver cirrhosis towards hepatocellular carcinoma in chronic hepatitis B and C infections: a review. Int J Mol Sci.

[14] Petropoulos S., Matthews S.G., Szyf M. (2014). Adult glucocorticoid exposure leads to transcriptional and DNA methylation changes in nuclear steroid receptors in the hippocampus and kidney of mouse male offspring. Biol Reprod.

[15] Wan E.S., Qiu W., Baccarelli A. (2012). Systemic steroid exposure is associated with differential methylation in chronic obstructive pulmonary disease. Am J Respir Crit Care Med.

[16] Taniguchi I., Iwaya C., Ohnaka K. (2017). Genome-wide DNA methylation analysis reveals hypomethylation in the low-CpG promoter regions in lymphoblastoid cell lines. Hum Genomics.

[17] Genotype-tissue expression - GTEx portal. https://gtexportal.org/home/.

[18] Rebouissou S., Nault J.C. (2020). Advances in molecular classification and precision oncology in hepatocellular carcinoma. J Hepatol.

[19] Cambiaghi V., Giuliani V., Lombardi S. (2012). TRIM proteins in cancer. Adv Exp Med Biol.

[20] Yi B., Tang C., Tao Y. (2020). Definition of a novel vascular invasion-associated multi-gene signature for predicting survival in patients with hepatocellular carcinoma. Oncol Lett.

[21] Xing M., Li P., Wang X. (2019). Overexpression of p62/IMP2 can promote cell migration in hepatocellular carcinoma via activation of the Wnt/β-catenin pathway. Cancers (Basel).

[22] Graumann R., Di Capua G.A., Oyarzún J.E. (2017). Expression of teneurins is associated with tumor differentiation and patient survival in ovarian cancer. PLoS One.

[23] Liu X., Rosenthal S.B., Meshgin N. (2020). Primary alcohol-activated human and mouse hepatic stellate cells share similarities in gene-expression profiles. Hepatol Commun.

[24] Lafuente E.M., van Puijenbroek A.A., Krause M. (2004). RIAM, an Ena/VASP and Profilin ligand, interacts with Rap1-GTP and mediates Rap1-induced adhesion. Dev Cell.

[25] Mo J., Zhao Y., Ao Z. (2020). Circ-APBB1IP as a prognostic biomarker promotes clear cell renal cell carcinoma progression through the ERK1/2 signaling pathway. Int J Med Sci.

[26] Li J., Zhou X., Liu X. (2019). Detection of colorectal cancer in circulating cell-free DNA by methylated CpG tandem amplification and sequencing. Clin Chem.

[27] Shen Q., Polom K., Williams C. (2019). A targeted proteomics approach reveals a serum protein signature as diagnostic biomarker for resectable gastric cancer. EBioMedicine.

[28] Zhu Z., Zhang F., Hu H. (2016). Integration of summary data from GWAS and eQTL studies predicts complex trait gene targets. Nat Genet.

[29] Jacques C., Baris O., Prunier-Mirebeau D. (2005). Two-step differential expression analysis reveals a new set of genes involved in thyroid oncocytic tumors. J Clin Endocrinol Metab.

[30] Sotomayor M., Gaudet R., Corey D.P. (2014). Sorting out a promiscuous superfamily: towards cadherin connectomics. Trends Cell Biol.

[31] Kim S.J., Jeong Y.T., Jeong S.R. (2020). Neural regulation of energy and bone homeostasis by the synaptic adhesion molecule calsyntenin-3. Exp Mol Med.

[32] Skawran B., Steinemann D., Weigmann A. (2008). Gene expression profiling in hepatocellular carcinoma: upregulation of genes in amplified chromosome regions. Mod Pathol.

[33] Ijaz B., Ahmad W., Das T. (2019). HCV infection causes cirrhosis in human by step-wise regulation of host genes involved in cellular functioning and defense during fibrosis: identification of bio-markers. Genes Dis.

[34] Schoggins J.W., Rice C.M. (2011). Interferon-stimulated genes and their antiviral effector functions. Curr Opin Virol.

[35] Zhao Y., Kang H., Ji Y. (2013). Evaluate the relationship between polymorphisms of OAS1 gene and susceptibility to chronic hepatitis C with high resolution melting analysis. Clin Exp Med.

[36] Lopez-Rodriguez R., Hernandez-Bartolome A., Borque M.J. (2017). Interferon-related genetic markers of necroinflammatory activity in chronic hepatitis C. PLoS One.

[37] Chen X., Steill J.D., Oomens J. (2010). Oxazolone versus macrocycle structures for Leu-enkephalin b2-b4: insights from infrared multiple-photon dissociation spectroscopy and gas-phase hydrogen/deuterium exchange. J Am Soc Mass Spectrom.

[38] Zhang Y., Yu C. (2020). Prognostic characterization of OAS1/OAS2/OAS3/OASL in breast cancer. BMC Cancer.

[39] Gomez-Skarmeta J.L., Modolell J. (2002). Iroquois genes: genomic organization and function in vertebrate neural development. Curr Opin Genet Dev.

[40] Zhu L., Dai L., Yang N. (2020). Transcription factorIRX5 promotes hepatocellular carcinoma proliferation and inhibits apoptosis by regulating the p53 signalling pathway. Cell Biochem Funct.

[41] Martorell O., Barriga F.M., Merlos-Suarez A. (2014). Iro/IRX transcription factors negatively regulate Dpp/TGF-beta pathway activity during intestinal tumorigenesis. EMBO Rep.

[42] Liu T., Zhang X., Yang Y.M. (2016). Increased expression of the long noncoding RNA CRNDE-h indicates a poor prognosis in colorectal cancer, and is positively correlated with IRX5 mRNA expression. Onco Targets Ther.

[43] Zou S., Wang C., Liu J. (2017). Sox12 is a cancer stem-like cell marker in hepatocellular carcinoma. Mol Cells.

[44] Huang W., Chen Z., Shang X. (2015). Sox12, a direct target of FoxQ1, promotes hepatocellular carcinoma metastasis through up-regulating Twist1 and FGFBP1. Hepatology.

[45] Sang X., Wu F., Wu D. (2020). Human hepatic cancer stem cells (HCSCs) markers correlated with immune infiltrates reveal prognostic significance of hepatocellular carcinoma. Front Genet.

[46] Yuan P., Meng L., Wang N. (2017). SOX12 upregulation is associated with metastasis of hepatocellular carcinoma and increases CDK4 and IGF2BP1 expression. Eur Rev Med Pharmacol Sci.

[47] Jiang T., Guan L.Y., Ye Y.S. (2017). MiR-874 inhibits metastasis and epithelial-mesenchymal transition in hepatocellular carcinoma by targeting SOX12. Am J Cancer Res.

[48] Zhang W., Liu K., Liu S. (2018). MicroRNA744 inhibits migration and invasion of hepatocellular carcinoma cells by targeting SOX12. Oncol Rep.

[49] Ke Y., Bao T., Zhou Q. (2017). Discs large homolog 5 decreases formation and function of invadopodia in human hepatocellular carcinoma via Girdin and Tks5. Int J Cancer.

[50] Wang D., Zhang Q., Li F. (2019). beta-TrCP-mediated ubiquitination and degradation of Dlg5 regulates hepatocellular carcinoma cell proliferation. Cancer Cell Int.

[51] Falade-Nwulia O., Suarez-Cuervo C., Nelson D.R. (2017). Oral direct-acting agent therapy for hepatitis C virus infection: a systematic review. Ann Intern Med.

[52] Rinaldi L., Nevola R., Franci G. (2020). Risk of hepatocellular carcinoma after HCV clearance by direct-acting antivirals treatment predictive factors and role of epigenetics. Cancers (Basel).

